# Anterior Tibial Vessel Turnover as an Alternative Recipient Strategy in Lower Extremity Free Flap Reconstruction

**DOI:** 10.3390/jcm15093448

**Published:** 2026-04-30

**Authors:** Young Jun Kim, Jun Mo Kim, Woo Young Choi, Ji Seon Cheon, Jeong Yeol Yang

**Affiliations:** 1 Department of Plastic and Reconstructive Surgery, Capital Corps, Republic of Korea Army, Incheon 21315, Republic of Korea; luck_kyj@naver.com; 2 Department of Plastic and Reconstructive Surgery, Chosun University Hospital, Gwangju 61453, Republic of Korea; moreword@csuh.co.kr (J.M.K.); jscheon@chosun.ac.kr (J.S.C.); jyyang@chosun.ac.kr (J.Y.Y.)

**Keywords:** tibial arteries, free tissue flaps, lower extremity, microsurgery, reconstructive surgical procedures

## Abstract

**Background/Objectives:** Reconstruction of complex lower extremity soft tissue defects remains challenging, particularly in the proximal and middle tibial regions, including the knee, where suitable recipient vessels are often limited due to prior trauma, infection, or surgical intervention. This study aimed to evaluate the feasibility and clinical applicability of anterior tibial vessel turnover as an alternative recipient vessel strategy in free flap reconstruction. **Methods:** A retrospective review was conducted of seven patients who underwent free flap reconstruction using anterior tibial vessel turnover as the recipient vessel between 2019 and 2024. Preoperative imaging was performed to assess vascular status and collateral circulation. Clinical data, including patient demographics, defect characteristics, flap parameters, and postoperative outcomes, were analyzed. **Results:** The mean patient age was 62.7 years (range, 38–86 years). Defects were primarily located in the proximal and middle tibial regions and were associated with trauma, postoperative infection, chronic osteomyelitis, or burn injury. The mean flap size was 137.4 cm^2^ (range, 49.5–280 cm^2^). All flaps survived, resulting in a flap survival rate of 100%, with no cases of total flap loss or re-exploration due to vascular compromise. One patient experienced partial flap loss, while no other flap-related complications were observed. Most patients achieved stable wound coverage and favorable functional recovery. **Conclusions**: Anterior tibial vessel turnover may serve as an alternative recipient vessel strategy for selected cases of complex lower extremity free flap reconstruction. This technique enables microvascular anastomosis in a more superficial and accessible field and expands reconstructive options in cases with compromised recipient vessels.

## 1. Introduction

Soft tissue defects of the lower extremity represent a persistent reconstructive challenge, particularly in the proximal and middle tibial regions, including the knee. These defects commonly result from trauma, chronic infection, oncologic resection, or postoperative complications and are frequently associated with extensive scar formation and compromised vascularity [1,2,3].

Reconstruction in this region requires durable and well-vascularized tissue coverage. Although local and regional flaps remain useful for small-to-moderate defects, their application is often limited in cases involving large defects or previously operated fields [2,4,5]. In such situations, free tissue transfer provides a reliable option by introducing healthy tissue from outside the zone of injury [6,7,8].

A critical determinant of success in free flap reconstruction is the selection of appropriate recipient vessels. However, in the proximal and middle tibial regions, recipient vessel selection can be challenging. Conventional recipient vessels, including the popliteal, genicular, and superficial femoral systems, are often located in relatively deep anatomical planes and may be compromised by prior trauma, infection, or surgical intervention [9,10,11,12,13]. These factors increase technical difficulty and may prolong operative time while raising the risk of anastomotic complications.

The anterior tibial vessels may represent an alternative recipient option because of their relatively constant anatomy and accessible course. However, careful patient selection and vascular assessment are essential when this strategy is considered. Published clinical data regarding anterior tibial vessel turnover as a recipient vessel strategy remain limited.

In this study, we present our expanded clinical experience with anterior tibial vessel turnover as a recipient vessel in lower extremity free flap reconstruction. We aim to evaluate its feasibility and clinical applicability across a range of challenging reconstructive scenarios.

## 2. Methods

### 2.1. Study Design and Patient Selection

This retrospective case series included seven consecutive patients who underwent lower extremity free flap reconstruction using anterior tibial vessel turnover as the recipient vessel between 2019 and 2024 at a single tertiary referral center. Patients were included when defects of the proximal or middle tibial region required free tissue transfer and conventional recipient vessels were considered limited or unsuitable because of deep location, scarring, prior trauma, infection, or previous surgical intervention. Patients with defects amenable to primary closure, skin grafting, or local/regional flap reconstruction were excluded.

### 2.2. Data Collection

Electronic medical records, operative reports, and clinical photographs were retrospectively reviewed. Collected variables included patient age, sex, diagnosis, etiology, comorbidities, defect location, previous surgery, flap type, flap size, pedicle length, exposed underlying structures, flap-related complications, and reconstructive outcomes.

Primary outcomes included flap survival, total or partial flap necrosis, vascular compromise requiring re-exploration, and achievement of stable wound coverage during the available follow-up period. Secondary descriptive variables included flap dimensions, pedicle length, recipient-site characteristics, and prior reconstructive history.

### 2.3. Preoperative Evaluation

All patients underwent preoperative clinical assessment with attention to wound condition, soft tissue quality, infection status, and reconstructive requirements. Serial debridement and infection control were performed when indicated based on clinical findings and microbiological results.

Computed tomography (CT) or magnetic resonance imaging (MRI) was used to evaluate defect extent and associated pathology such as osteomyelitis. In addition, CT angiography was routinely performed to assess the anterior tibial vessels, distal runoff, vessel patency, and collateral circulation. The turnover technique was considered only when adequate alternative perfusion through the posterior tibial and/or peroneal arterial systems or robust collateral flow was confirmed.

### 2.4. Surgical Technique and Postoperative Management

All reconstructions were performed using free tissue transfer, most commonly an anterolateral thigh (ALT) flap. Perforators were identified preoperatively using a handheld Doppler. Through a longitudinal incision along the lateral tibial aspect, the anterior tibial artery and venae comitantes were exposed between the tibialis anterior and extensor musculature.

**Figure 1:**
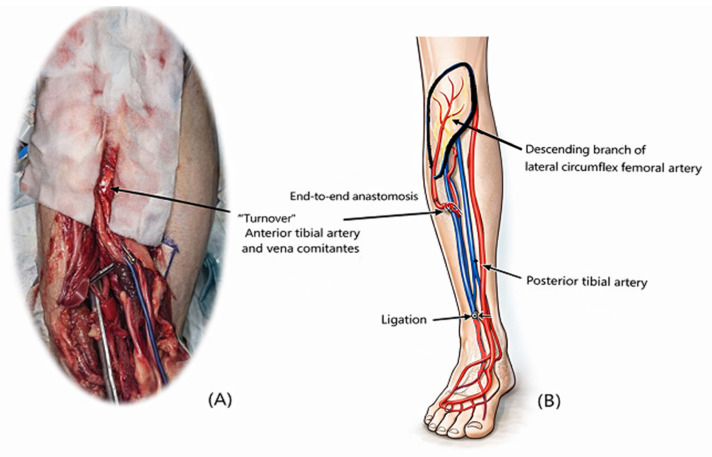
Schematic illustration of anterior tibial vessel turnover technique. (**A**) Intraoperative picture of turnover anterior tibial artery and vena comitantes. (**B**) Diagram demonstrating mobilization and turnover of the anterior tibial vessels.

The vessels were dissected to sufficient length for mobilization, with ligation of side branches as needed, and then turned over toward the defect. Microvascular anastomosis was performed in an end-to-end fashion (Figure 1). The flap was inset without tension, and the pedicle was positioned to avoid kinking or compression. Intraoperative flap perfusion was confirmed by clinical examination, including flap color, capillary refill, and Doppler signals.

Postoperatively, flap monitoring was performed hourly during the first 48 h and every 4 h thereafter using serial clinical examination, pin-prick testing, and handheld Doppler assessment. Routine postoperative management included intravenous prostaglandin E1, prophylactic anticoagulation with low-molecular-weight heparin, and early mobilization according to institutional protocol.

### 2.5. Ethical Considerations

This study was conducted in accordance with the Declaration of Helsinki. Institutional review board approval was waived because of the retrospective nature of the study, and written informed consent was obtained from all patients. OpenAI ChatGPT (GPT-4, OpenAI, San Francisco, CA, USA) was used exclusively for language editing and readability enhancement. All scientific content, interpretation, and final manuscript decisions were performed and verified by the authors.

## 3. Results

A total of seven patients were included in this study. The mean age was 62.7 years (range, 38–86 years). The etiologies included trauma, postoperative infection, chronic osteomyelitis, and burn injury. Most patients had undergone prior surgical interventions. The defects were located in the proximal and middle tibial regions. The mean flap size was 137.4 cm^2^ (range, 49.5–280 cm^2^) (Table 1). Representative cases are presented below to illustrate the surgical technique and range of clinical indications.

All flaps survived, yielding a flap survival rate of 100%. No cases of total flap loss were observed. One patient experienced partial flap loss. No cases required re-exploration due to vascular compromise. Most patients achieved stable wound coverage without additional reconstructive procedures. Functional recovery, including ambulation and knee range of motion greater than 90°, was observed in applicable cases. All patients achieved stable wound coverage during the available postoperative follow-up period, although follow-up duration was not uniform across cases.

### 3.1. Case 1 (Patient No. 1)

**Figure 2:**
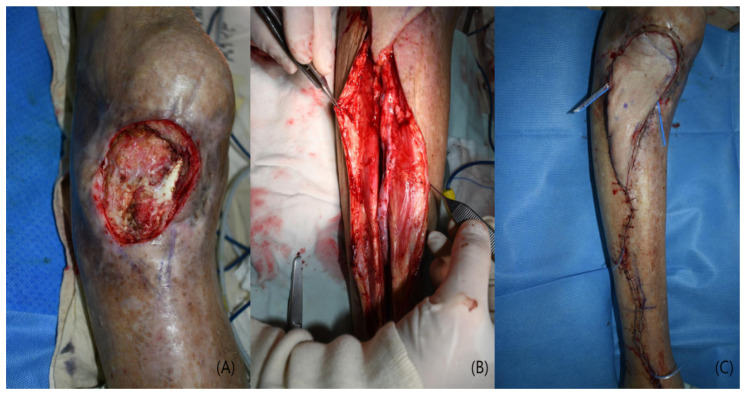
Case 1 (Patient No. 1) intraoperative findings. (**A**) Soft tissue defect after debridement. (**B**) End-to-end microvascular anastomosis using turnover anterior tibial vessels. (**C**) Immediate postoperative flap inset.

**Figure 3:**
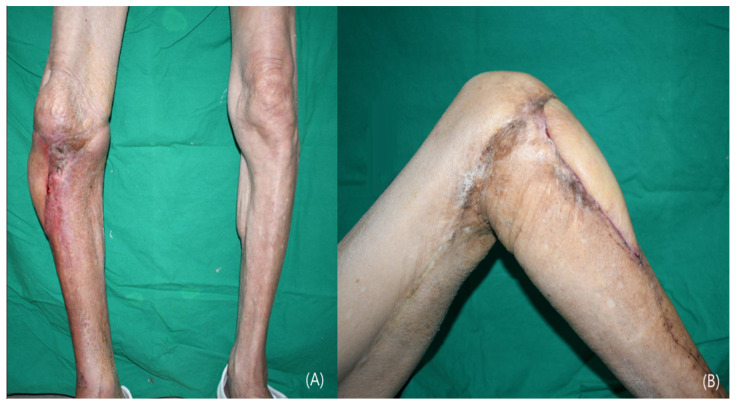
Case 1 (Patient No. 1) representative postoperative outcomes at 3 months. (**A**) Weight-bearing standing position. (**B**) Knee flexion greater than 90 degrees.

An 86-year-old man with a history of hypertension presented with a chronic osteomyelitis of the proximal tibia following trauma. The defect involved exposure of bone and tendon. After serial debridement and infection control, reconstruction was performed using an ALT free flap. The anterior tibial vessels were identified, mobilized, and turned over to serve as recipient vessels (Figure 2). Microvascular anastomosis was performed in an end-to-end fashion. The flap measured 15 × 6 cm, and the donor site was closed primarily. The postoperative course was uneventful, with complete flap survival and no complications. At 3-month follow-up, the patient achieved stable wound coverage and was able to ambulate with knee flexion greater than 90° (Figure 3).

### 3.2. Case 2 (Patient No. 2)

**Figure 4:**
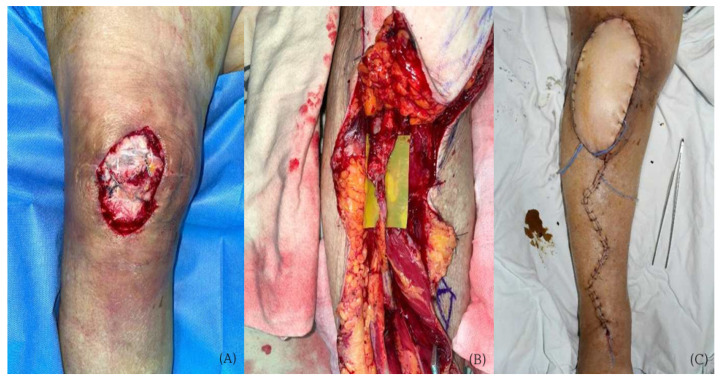
Case 2 (Patient No. 2) representative intraoperative findings. (**A**) Soft tissue defect after debridement. (**B**) End-to-end microvascular anastomosis using turnover anterior tibial vessels. (**C**) Immediate postoperative flap inset.

A 76-year-old woman with hypertension and angina developed a postoperative infection following open reduction and internal fixation for fractures involving the proximal tibia and patella. The defect was characterized by exposure of the bone and orthopedic implant. After adequate wound bed preparation, free flap reconstruction was performed using an ALT flap. The anterior tibial vessels were used as recipient vessels with a turnover technique (Figure 4). The postoperative course was uneventful, with complete flap survival and no flap-related or systemic complications.

### 3.3. Case 3 (Patient No. 4)

**Figure 5:**
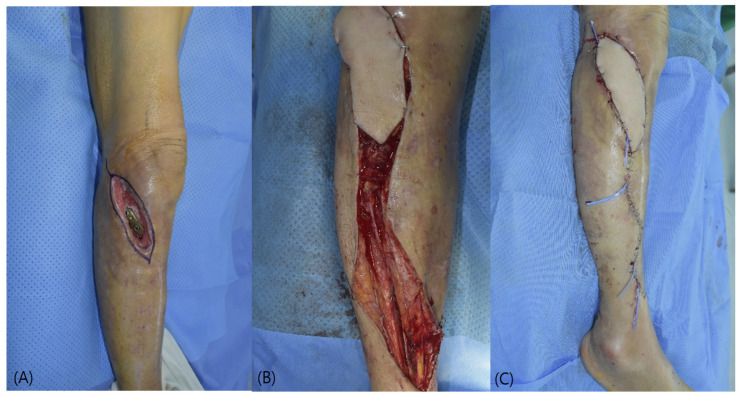
Case 3 (Patient No. 4) intraoperative findings. (**A**) Soft tissue defect of the proximal tibia with exposed bone and orthopedic hardware after debridement. (**B**) End-to-end microvascular anastomosis using turnover anterior tibial vessels. (**C**) Immediate postoperative flap inset.

An 80-year-old woman with a history of coronary artery disease presented with a postoperative infection of the proximal tibia following open reduction and internal fixation. The defect was characterized by exposure of bone and orthopedic hardware. After appropriate wound bed preparation, including debridement and infection control, reconstruction was performed using an ALT free flap. The anterior tibial vessels were identified and used as recipient vessels with a turnover technique (Figure 5). Microvascular anastomosis was performed in an end-to-end fashion. The flap measured 11 × 4.5 cm, and the pedicle length was 10 cm. The postoperative course was uneventful, with complete flap survival and no flap-related complications.

### 3.4. Case 4 (Patient No. 5)

**Figure 6:**
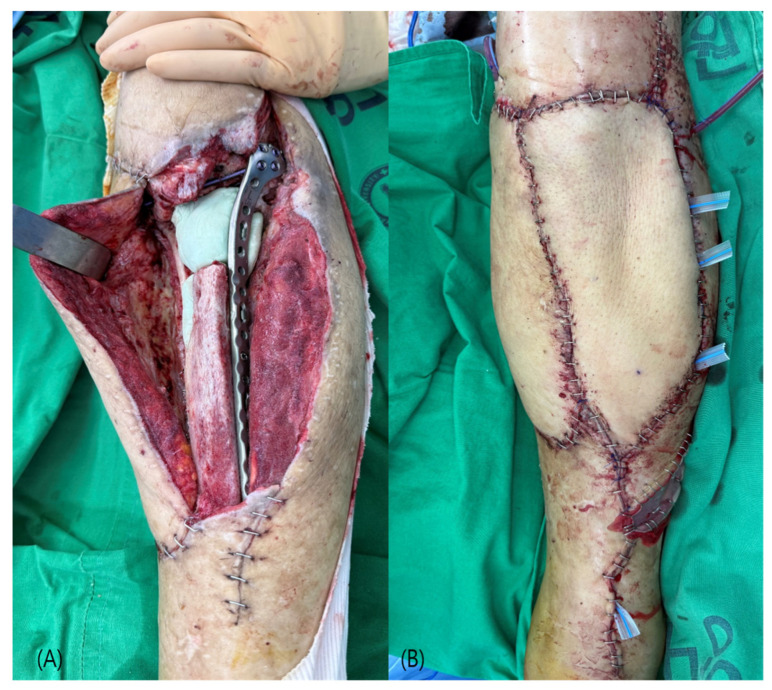
Case 4 (Patient No. 5) intraoperative findings. (**A**) Large soft tissue defect of the proximal tibia with exposed bone and fixation hardware after debridement. (**B**) Immediate postoperative flap inset following anterolateral thigh free flap coverage.

A 52-year-old man with a history of hypertension presented with chronic osteomyelitis of the proximal tibia. The patient had previously undergone open reduction and internal fixation, followed by a latissimus dorsi free flap reconstruction at another institution. Despite prior treatment, persistent infection and soft tissue breakdown resulted in a complex defect with exposure of bone and fixation hardware. After serial debridement and adequate infection control, reconstruction was performed using an ALT free flap. The anterior tibial vessels were identified, mobilized, and turned over to serve as recipient vessels (Figure 6). Microvascular anastomosis was performed in an end-to-end fashion in a superficial and accessible field. The flap measured 20 × 14 cm, and the donor site was closed primarily. The postoperative course was uneventful, with complete flap survival and no complications. Stable soft tissue coverage was achieved without the need for additional reconstructive procedures.

### 3.5. Case 5 (Patient No. 6)

**Figure 7:**
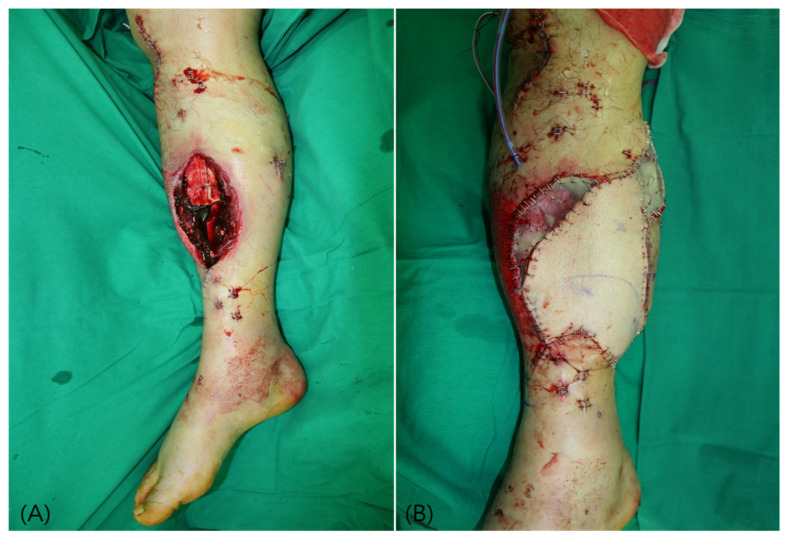
Case 5 (Patient No. 6) intraoperative findings. (**A**) Soft tissue defect of the middle tibia with exposed bone after debridement. (**B**) Immediate postoperative flap inset following anterolateral thigh free flap coverage.

A 41-year-old man presented with an open tibial fracture of the middle tibia caused by trauma. The defect involved exposure of bone, requiring soft tissue coverage. Following initial debridement and stabilization, reconstruction was performed using an ALT free flap. The anterior tibial vessels were dissected, mobilized, and turned over to serve as recipient vessels. Microvascular anastomosis was completed in an end-to-end manner. The flap measured 16 × 10 cm, with a pedicle length of 10 cm. The postoperative course was uneventful, with successful flap survival and stable wound coverage (Figure 7).

## 4. Discussion

Reconstruction of large soft tissue defects in the proximal and middle tibial regions, including the knee, remains a significant challenge in reconstructive surgery [2,14]. Various surgical techniques have been employed to address these defects, and in many cases, they can be managed using local tissues, including primary closure, skin grafting, local advancement flaps, or axial pattern muscle flaps [15].

However, large defects resulting from trauma, chronic infection, oncologic resection, or post-burn contracture release are often associated with insufficient pliable and well-vascularized tissue, making reliable wound healing difficult [3]. This is primarily due to compromised blood and lymphatic circulation, as well as extensive scar formation. In such cases, reconstruction using tissue transferred from outside the zone of injury is essential [6,7,8].

The reverse-flow (distally based) ALT flap has been widely used for knee reconstruction, offering advantages such as minimal donor-site morbidity, a long pedicle, and sufficient tissue volume [16]. Nevertheless, it is limited by its arc of rotation and carries a risk of venous congestion due to reversed blood flow and venous valve resistance [6,14].

Free tissue transfer provides a reliable option for reconstruction of large defects, allowing transfer of various tissue components and delivery of well-vascularized tissue to compromised recipient sites [7,8]. However, successful free flap reconstruction critically depends on the selection of appropriate recipient vessels [11,12,13]. In the proximal and middle tibial regions, recipient vessel selection remains challenging due to anatomical and pathological factors that may compromise vessel availability and quality [2,9,10,11,12,13].

Traditional recipient vessels around the knee, such as the popliteal, genicular, or superficial femoral vessels, are often located in deeper anatomical planes and require extensive dissection, which can increase operative time and technical complexity [11,12]. In addition, these vessels are frequently affected by fibrosis, intimal injury, or scarring related to prior trauma, infection, or surgical interventions, which may compromise vessel quality and increase the risk of thrombosis. Their limited mobility and deep location can also make pedicle routing more challenging and restrict the working space for microvascular anastomosis.

Alternative strategies, including the use of distal recipient vessels or vein grafts, have been described; however, these approaches may be associated with increased risk of thrombosis, prolonged operative time, and technical difficulty [15]. In contrast, anterior tibial vessel turnover allows for the use of a relatively superficial and anatomically consistent vascular pedicle, enabling direct anastomosis without the need for vein grafting. In contrast, anterior tibial vessel turnover allows the use of a relatively superficial and anatomically consistent vascular pedicle, enabling direct anastomosis without vein grafting in selected cases.

Previous studies have emphasized the importance of selecting recipient vessels outside the zone of injury to improve flap survival and reduce complications [6,7]. In this context, the anterior tibial vessel turnover technique aligns well with this principle by facilitating anastomosis in a healthier vascular environment. Although direct comparative studies are limited, our clinical outcomes support the feasibility and safety of this approach in complex reconstructive scenarios.

**Figure 8:**
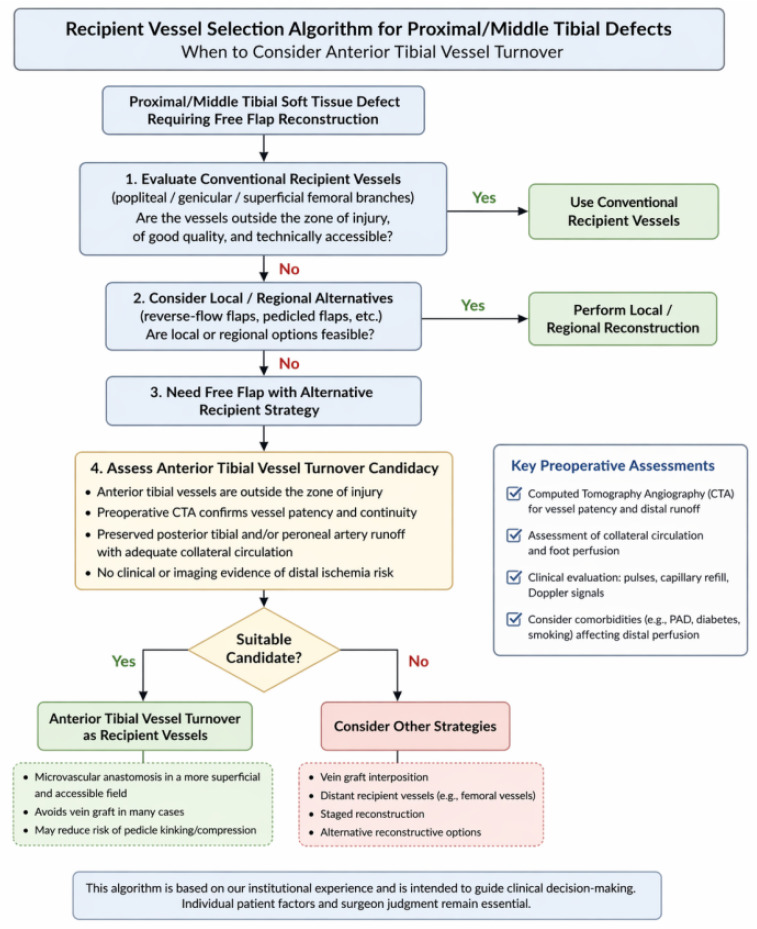
Proposed practical framework for recipient vessel selection in free flap reconstruction of proximal and middle tibial soft tissue defects. Anterior tibial vessel turnover may be considered in selected patients when conventional recipient vessels are unavailable or unsuitable and adequate distal perfusion is confirmed.

In the present series, anterior tibial vessel turnover was considered when conventional recipient vessels were unavailable, deeply located, scarred, or unsuitable because of prior trauma, infection, or previous surgery. Preoperative CT angiography was routinely performed to evaluate distal runoff, vessel patency, and collateral circulation, and this strategy was considered only when adequate vascular reserve was confirmed. Intraoperative assessment of vessel quality, distal perfusion, and technical accessibility also contributed to final decision-making. To improve practical applicability, we additionally propose a simplified recipient vessel selection framework for proximal and middle tibial defects requiring free flap reconstruction (Figure 8). This framework was retrospectively derived from our institutional experience and is intended to assist clinical judgment rather than replace surgeon discretion.

All flaps survived, with no cases of total flap loss or re-exploration due to vascular compromise, although one patient experienced partial flap loss. These findings support the technical feasibility of this technique, even in challenging clinical situations.

One of the key technical advantages of anterior tibial vessel turnover is that it allows microvascular anastomosis to be performed in a relatively superficial and accessible field. Conventional recipient vessels around the knee, such as the popliteal or genicular systems, are often located in deeper planes [9,10]. In contrast, the anterior tibial vessels can be mobilized and repositioned outside the zone of injury, enabling tension-free anastomosis. Their relatively constant anatomy may also facilitate identification and dissection, particularly in cases with severe scarring or distorted local anatomy.

Another potential advantage is improved pedicle geometry. Because the recipient vessels can be positioned closer to the defect, pedicle routing may be simplified, and the likelihood of pedicle kinking or compression may be reduced. From a technical perspective, dissection of the anterior tibial vessels can be initiated from the distal leg or ankle region and extended proximally, where the vessels are often more superficial and anatomically consistent.

Despite these potential advantages, anterior tibial vessel turnover has inherent limitations. The technique requires meticulous dissection with ligation of multiple side branches, which may increase operative time. In addition, this procedure necessitates sacrifice of the anterior tibial artery, one of the major axial vessels of the leg. Therefore, it should not be routinely applied and must be reserved for carefully selected patients with preserved posterior tibial/peroneal circulation or robust collateral flow. Careful preoperative assessment of distal perfusion using CT angiography and Doppler evaluation is essential before employing this technique [15,17]. Preoperative imaging demonstrated adequate alternative arterial runoff in all patients, and no postoperative signs of distal foot ischemia were identified. Furthermore, pedicle-related parameters, operative efficiency, and comparative outcomes were not formally quantified in the present study.

From a clinical standpoint, this technique may be particularly useful in limb salvage situations where conventional recipient vessels are unavailable or unsuitable. In such cases, anterior tibial vessel turnover provides a practical alternative that expands reconstructive options for complex lower extremity defects.

This study has several limitations. The sample size was small, and the retrospective design may introduce selection bias. Because this was a retrospective case series of selected patients, cases in which this strategy was considered but not ultimately used were not systematically analyzed. In addition, follow-up duration was not standardized across patients, which limits assessment of long-term durability and functional outcomes. Further studies with larger patient cohorts and longer follow-up periods are required to validate the generalizability and long-term reliability of this technique.

## 5. Conclusions

Anterior tibial vessel turnover may provide an alternative recipient vessel option in selected cases of complex lower extremity reconstruction. In this small retrospective series, the technique was technically feasible and achieved favorable short-term outcomes. Further comparative studies are required to define its optimal role.

## Figures and Tables

**Table 1:** Patient data.

No.	Age (y)	Sex	Diagnosis	Etiology	Comorbidity	Location	Previous Surgery	Flap Size (cm^2^)	Pedicle Length (cm)	Flap-Related Complication	Exposure	Reconstruction
1	86	M	Chronic osteomyelitis	Trauma	HTN	Proximal tibia, patella	None	15 × 6	13	None	Bone, tendon	ALT flap
2	76	F	Postoperative infection	Trauma	HTN, angina	Proximal tibia, patella	ORIF	15 × 7	10	None	Bone, implant	ALT flap
3	38	M	Postoperative infection	Trauma	DM	Distal femur, patella	ORIF	20 × 8	10	Partial flap loss	Bone, tendon	TDAP flap
4	80	F	Postoperative infection	Trauma	CAD	Proximal tibia	ORIF	11 × 4.5	10	None	Bone, implant	ALT flap
5	52	M	Chronic osteomyelitis	Chronic infection	HTN	Proximal tibia	ORIF, Latissimus dorsi free flap	20 × 14	12	None	Bone, implant	ALT flap
6	41	M	Open tibial fracture	Trauma	None	Middle tibia	None	16 × 10	10	None	Bone	ALT flap
7	66	F	Flame burn injury	Burn	DM, HTN	Proximal tibia, patella	STSG	13 × 9	12	None	Bone	ALT flap

Abbreviations: ALT, anterolateral thigh; CAD, coronary artery disease; DM, diabetes mellitus; HTN, hypertension; ORIF, open reduction and internal fixation; STSG, split-thickness skin graft; TDAP, thoracodorsal artery perforator.

## Data Availability

Data sharing is not applicable to this article, as no new data were created or analyzed.

## Abbreviations

The following abbreviations are used in this manuscript:

ALT	Anterolateral thigh
CT	Computed tomography
MRI	Magnetic resonance imaging
